# Dimensional Structure of and Variation in Anthropomorphic Concepts of God

**DOI:** 10.3389/fpsyg.2018.01425

**Published:** 2018-08-10

**Authors:** Nicholas J. Shaman, Anondah R. Saide, Rebekah A. Richert

**Affiliations:** ^1^Department of Psychology, University of Houston–Clear Lake, Houston, TX, United States; ^2^Educational Psychology, University of North Texas, Denton, TX, United States; ^3^Department of Psychology, University of California, Riverside, Riverside, CA, United States

**Keywords:** anthropomorphism, religious cognition, cognitive science of religion, religiosity, supernatural agents

## Abstract

When considering other persons, the human mind draws from folk theories of biology, physics, and psychology. Studies have examined the extent to which people utilize these folk theories in inferring whether or not God has human-like biological, physical, and psychological constraints. However, few studies have examined the way in which these folk attributions relate to each other, the extent to which attributions within a domain are consistent, or whether cultural factors influence human-like attributions within and across domains. The present study assessed 341 individuals’ attributions of anthropomorphic properties to God in three domains (psychological, biological, and physical), their religious beliefs, and their engagement in religious practices. Three Confirmatory Factor Analyses tested hypothetical models of the underlying structure of an anthropomorphic concept of God. The best fitting model was the “Hierarchical Dimensions Concept,” the analyses indicated one overall dimension of anthropomorphism with three sub-domains. Additionally, participants’ religiosity was negatively related to attributing human-like psychological properties to God, suggesting that the more people engage with their religion, the less they think about God as having a ‘human-like’ mind. Religiosity was positively related to individual consistency scores in the biological domain. In other words, greater religiosity was related to less consistent answers about God’s biological properties. As a result, the findings of the current study also suggest that individuals do not just vary between each other in how much they anthropomorphize God, but additionally, variation exists in the type of anthropomorphic reasoning used within an individual person’s concept of God.

## Introduction

The tendency for humans to anthropomorphize non-human entities across the life course has been well documented (e.g., Heiphetz et al., 2016; Nyhof and Johnson, 2017). Rather than focus on the generalized tendency to anthropomorphize non-human entities and objects by attributing to them agency and mental states, the current study examines the extent to which anthropomorphizing supernatural beings, such as God, occurs across domains and for uniquely human-like psychological processes. There is considerable evidence suggesting that applying human-like traits to non-human entities, like animals, computers, shapes, and supernatural beings, may constitute an innate cognitive bias, a bias that is common among all human minds (Guthrie, 1993; Dacey, 2017). In practice, an anthropomorphic bias leads people to make inferences about entities using their concept of “human" or “agent," rather than based on direct observable evidence from that entity (Rottman and Kelemen, 2012). Both children and adults will apply human-like traits even to geometric figures (e.g., triangles, squares) if those figures seem to move in systematized (i.e., patterned) ways (Csibra et al., 1999). Although some researchers are unpacking the nature of individual differences in the tendency to anthropomorphize (e.g., Waytz et al., 2010; Severson and Lemm, 2016), studies aiming to unpack the underlying structure of anthropomorphic concepts and relate that structure to folk reasoning in domains of psychology, physics, and biology, have been limited. The current study leverages the fact that concepts of unobservable, supernatural agents (e.g., God) are represented across human cultures and in human minds to examine the underlying dimensional structure of anthropomorphic attributions to God.

By studying how individuals make anthropomorphic inferences about unobservable, supernatural agents the current study simultaneously addresses three often understudied aspects of anthropomorphizing from the existing literature. First, the current study examines the attributions of human-like, rather than general, psychological properties to God. Although most studies of anthropomorphic reasoning consider it to be driven by folk psychological cognitive processes, anthropomorphic attributes are often conflated with attributions of agency (Epley et al., 2007). There are however, important differences between attributions of agency (self-propelled movement and having goal-directed actions) and mentality [goal-directed actions (i.e., agency) that are driven by internal thoughts, beliefs, emotions, perceptions, and desires] (Barrett, 2008); and there are further distinctions that are associated with *human-like* agency and mentalizing. However, studies that focus on the mentalizing attributions to non-human agents and objects typically focus on mental states that are also regularly associated with non-human entities (i.e., animals), such as having thoughts, desires, and perceptions (Epley et al., 2007; Shtulman and Lindeman, 2016).

Less commonly studied are inferences individuals make about whether non-human entities have *human-like* cognitions, such as the ability to pretend. Asking individuals whether they would apply specific, human-like psychological states to non-human entities can highlight the extent to which anthropomorphic reasoning about spiritual agents involves more than general attributions of agency or mentalizing, but rather attributions of *human-like* agency or mentalizing.

Second, studies of anthropomorphism must contend with the fact that anthropomorphizing involves the coordination of different inputs and cognitive processes, inputs based on direct observation or experience, in conjunction with the use of anthropomorphic reasoning. In research, the difficulty in delineating the role of anthropomorphic reasoning in concept formation arises when the information coming from these inputs overlaps. For example, a computer is a physical object that must conform to many of the same physical laws as a human body. Thus, a study that identifies that people attribute human-like physical traits to a computer would not be able to disentangle if that attribution is based in direct observation of computers conforming to physical laws, generally speaking, or to the inferences made that a computer must conform to the same physical laws as a person. Studying anthropomorphic attributions to an unobservable entity such as God, removes this confound, reflecting anthropomorphism through inference, rather than through direct experience (e.g., people cannot see if God has a body, they must infer if God has a body).

Third, studies of anthropomorphic reasoning often only assess the psychological attributions people make about non-human entities. However, humans are conceptualized as having biological and physical attributes as well. The focus on psychological attributes exclusively is due to the fact that the non-human entities examined have biological and/or physical attributes of their own, independent of any anthropomorphic inferences. For example, a person may infer that a dog needs to eat and cannot pass through walls, but that inference is not made because of any anthropomorphic reasoning. Asking individuals to make inferences about a non-human entity that does not have a corporeal form (according to religious or cultural messages) provides an opportunity to examine anthropomorphic reasoning beyond the psychological realm.

The current study of God concepts addresses each of these understudied aspects and delineates the structural nature of anthropomorphic concepts. Overall, the present study sought to determine: (a) the underlying structure of individuals’ anthropomorphic concept of God, (b) whether there are cultural and experiential predictors of that structure, and (c) whether individuals are consistent in how they anthropomorphize the different sub-domains of concepts of God. To examine these research questions, the present study assessed individuals’ attribution of anthropomorphic properties to God in three domains (i.e., psychological, biological, and physical), their religious beliefs, and their engagement in religious practices. Within each domain of human-like traits, participants were asked about characteristics of humans that would differentiate humans from an omniscient, omnipotent, and omnipresent explicit concept of God (e.g., humans can forget, God cannot forget). The primary contributions of the current study include an analysis of how these domains relate to one another and an exploration of the experiential (e.g., religious belief and participation) and personal (e.g., belief in God) factors that contribute to individual differences in anthropomorphizing across the three domains.

## Anthropomorphic Reasoning

According to Epley et al. (2007), there are at least three separate factors that contribute to the tendency to anthropomorphize: (a) people use concepts of agency to reason about non-human entities, (b) people are motivated to understand the behavior of non-human entities, and (c) people are socially motivated to seek social contact. The first factor, the tendency to use of concepts of agency to reason about non-human entities, is the most heavily researched, particularly in the cognitive science of religion (e.g., Rottman and Kelemen, 2012; Heiphetz et al., 2016). From this perspective, when a person is reasoning about a non-human entity, that person conceptualizes that entity as an intentional actor that wants to effect some change upon the world. A set of assumptions can follow once an entity has been characterized as an intentional actor, including the assumption that the entity has mental states (including knowledge, emotions, and/or desires) that drive actions.

However, humans are not just conceptualized as intentional actors, but also as biological entities that obey the laws of physics. When making inferences about human beings, people do not only use their folk-psychological reasoning but use their folk-biological and folk-physical reasoning as well. A concept of a human is an entity that has mental states that drive action, but also has biological processes and obeys the laws of physics. However, there is debate as to whether people make anthropomorphic inferences based solely on their folk-psychological reasoning or their concept of ‘human’ (Rottman and Kelemen, 2012). If people only apply their folk-psychological reasoning to non-human entities, they would only make assumptions of agency and mentality. If people use their concept of ‘human,’ which includes all three domains of folk knowledge, when reasoning about a non-human entity, they would also make assumptions of growth and physicality. When engaging in anthropomorphic reasoning, thinking that a dog can have human-like mental states is just as anthropomorphic as thinking that God has a biological body. However, studies have suggested there are circumstances in which people are more or less likely to apply folk-psychological reasoning or their concept of ‘human’ to non-human entities.

More specifically, studies have begun to document extensive variation in the ways in which people anthropomorphize. Waytz et al. (2010) examined individual differences in people’s tendency to anthropomorphize, creating and validating the Individual Differences in Anthropomorphism Questionnaire (IDAQ). Using this measure, Waytz et al. (2010) found individual differences in how much people anthropomorphize non-human entities and found these differences to be stable over time. When spiritual entities were among the non-human entities participants were asked to consider, spiritual entities loaded on a separate factor than animals and non-animals (technologies). Waytz et al. (2010) primarily operationalized anthropomorphism as the extent to which people attribute mental states to non-human entities (e.g., mind, free will, consciousness). Regarding the spiritual entities in particular, participants did not discriminate anthropomorphic and non-anthropomorphic traits from each other; Waytz et al. (2010) interpreted this finding to mean the measure more likely was a measure of belief in spiritual agents rather than anthropomorphism of spiritual agents.

Without the spiritual agents, the IDAQ had two underlying factors: anthropomorphism of animals and anthropomorphism of non-animals (Waytz et al., 2010). Waytz et al. (2010) found that these two factors were related in such a way as to suggest two factors within a superordinate tendency to anthropomorphize, with animal anthropomorphizing loading more strongly than non-animal anthropomorphizing. By collapsing the two factors together for a dispositional trait measure of anthropomorphism, the researchers found that increases in anthropomorphic reasoning are related to moral judgments of non-human entities, environmental concern, and trust in technological agents (i.e., computers and robots) (Waytz et al., 2010). In other words, the more an individual anthropomorphizes an agent, the greater reported belief that the agent deserves moral regard, moral care, and is trusted. This body of work indicates that anthropomorphizing differs both by individual but also by entity (human versus different types of non-humans). Regarding spiritual entities, the conflation in the IDAQ of anthropomorphizing with belief in spiritual entities suggests individual differences in anthropomorphizing spiritual entities involves more than reasoning about just their agency.

### Anthropomorphic Reasoning About God

Although the tendency to anthropomorphize non-human entities is seen as a universal and innate behavior, an individual’s cultural context influences which non-human entities are anthropomorphized and how those entities are anthropomorphized. Nowhere is this clearer than in the conceptualization of supernatural beings (Heiphetz et al., 2016). In cultures across the world, people often conceptualize supernatural beings, such as gods, spirits, and ghosts, as having minds or mental states that are similar to humans (e.g., Knight et al., 2004). A deity can have human-like emotions, a spirit can act intentionally, and a ghost can have mental limitations, such as ignorance (Nyhof and Johnson, 2017).

Given the interest in the current study on the extent to which adults make anthropomorphic inferences about God, the current study measured explicit concepts of God. However, it should be noted that people do not just anthropomorphize supernatural beings explicitly in stories or religious practices. In fact, although supernatural beings are often assigned special mental, biological, or physical properties that distinguish them from humans explicitly (i.e., consciously) (Boyer, 2001), people implicitly (i.e., non-verbally, unconsciously) conceptualize these beings as having human-like properties as well (Heiphetz et al., 2016). In other words, while adults may explicitly reason about God in non-anthropomorphic ways (i.e., God is omniscient), they may implicitly conceive of God as human-like, with human limitations and needs (Barrett and Keil, 1996; Shtulman and Lindeman, 2016).

One method for tapping into participants’ implicit anthropomorphic reasoning while asking explicit questions is to ask participants to rate their certainty about whether or not God has certain human-like traits (Richert et al., 2017). For example, in a study measuring the relation between parents’ anthropomorphic attributions to God and children’s differentiation of God’s mind and human minds, parents indicated their certainty that God had specific psychological (e.g., could forget), biological (e.g., could get sick), or physical (e.g., could get wet in the rain) limitations. Although parents globally answered no to these questions, Muslim parents were significantly more certain that God did not have these kinds of anthropomorphic traits than Protestant Christian, Roman Catholic, or Religiously Non-Affiliated parents (Richert et al., 2017).

Within the area of the cognitive science of religion, researchers have taken at least two approaches to the study of anthropomorphic concepts of the Judeo-Christian God. The first approach has been to explore how individuals conceptualize God’s mind and knowledge, as compared to that of other entities (e.g., Barrett et al., 2001; Knight et al., 2004; Makris and Pnevmatikos, 2007; Lane et al., 2012; Richert et al., 2017). These studies of applying folk-psychological reasoning to concepts of God have indicated support that some (primarily Christian and Non-Affiliated) children think of God has having human-like mental states and limitations, whereas other (primarily Muslim) children do not.

The second approach has examined the degree to which individuals reason that God has human-like properties in all three folk domains: psychological, biological, and physical properties (e.g., Shtulman, 2008; Shtulman and Lindeman, 2016). Studies that examined each domain separately suggest people attribute more psychological properties to God than biological properties. Shtulman (2008) examined how adults attributed various properties to God and fictional beings (like fairies and vampires). On average, participants stated that God and the fictional beings had more psychological traits than either biological or physical traits. These findings suggest that people apply more folk-psychological reasoning to God than folk-biological or folk-physical reasoning.

Extending this research, Shtulman and Lindeman (2016) examined how adults attributed psychological and biological and physical traits to God. In their study, the psychological traits were related to agency and mentality (e.g., having beliefs, desires, intentions, emotions, and the ability to perceive). The biological traits were related to biological processes (e.g., breathing, eating, aging, becoming ill) and biological organs (e.g., heart, brain, bones). The physical traits related to existing as a physical entity in the world that was subject to the laws of physics (e.g., exerts force, has weight). Consistent with the previous findings, adults attributed more psychological traits to God than biological or physical traits. Shtulman and Lindeman (2016) noted that despite the participants not attributing many biological or physical traits to God, the mean levels were not zero. Thus, adults did utilize their folk-biological and folk-physical reasoning to conceptualize God, just not in the same way as their folk-psychological reasoning.

### The Role of Religious Belief and Experience

As noted above, anthropomorphizing of culturally specific supernatural agents should be shaped by and responsive to the cultural context (Heiphetz et al., 2016). Support for this assertion has emerged in research into the influence of belief in God and religious exposure on anthropomorphizing of God. Shtulman and Lindeman (2016) found that measures of religiosity were related to attributions of human-like properties of God. Religiosity was measured with a 16-item questionnaire on daily spirituality, positive religious coping, participation in public and private forms of worship, and self-reported religiosity. Participants who reported higher levels of religiosity also were more likely to attribute psychological and physiological properties to God (although attributions of physiological properties to God was lower than attributions of psychological properties).

The positive nature of these correlation patterns indicates potential concern about confounding participants’ belief in God with attributions they make to God. For example, Willard and Norenzayan (2013) found that variation in a person’s ability to reason about mental-states (i.e., theory-of-mind) was linked to belief in God. Interestingly however, belief in God was unrelated to the general tendency to engage in anthropomorphic reasoning as measured by the IDAQ. This suggests that a person does not necessarily need to believe in the existence of God in order to hold anthropomorphic concepts of God. Additionally, Waytz et al. (2010) found that measures of anthropomorphizing God that focus on attributions of agency and mentality may conflate belief in God with attributing any traits at all to God.

Studies in the development of anthropomorphizing of God have suggested that religious factors other than belief in God may additionally relate to individuals’ anthropomorphizing. For example, children of parents who believed the actions of prayer serve a ritualized, communicative function were more anthropomorphic in their concepts of God than children of parents who believed that the actions of prayer were there to promote internal reflection (Richert et al., 2016).

Recent studies have also documented differences in anthropomorphic reasoning about God by religious tradition. In one study, Muslim children and parents anthropomorphized God significantly less than Protestant Christian and Catholic children and parents (Richert et al., 2016). In a study with children from Latter-Day Saints and mainstream Christian backgrounds, children demonstrated an understanding that supernatural agents like God, have special mental properties; and the religious traditions that those children were from, influenced that understanding (Nyhof and Johnson, 2017). In another study, Hindu adults were more likely than Protestant Christian adults to associate physiological traits to God (Shtulman and Lindeman, 2016). To assess the varying influences religious beliefs and practices may have on anthropomorphic reasoning about God, the current study incorporated measures of belief in God and the soul, participation in religious activities, and participation specifically in religious rituals.

## Research Questions

In summary, previous research on anthropomorphic reasoning has found that people use their concept of agency to make inferences about non-human entities (Epley et al., 2007). Between people, however, there is considerable variation in the tendency to anthropomorphize (Waytz et al., 2010; Severson and Lemm, 2016). In order to separate the role that cognitive processes and direct experience play in anthropomorphizing non-human entities, researchers have examined how people anthropomorphize God, a non-observable, non-human entity (e.g., Barrett and Keil, 1996; Barrett et al., 2001; Shtulman, 2008; Heiphetz et al., 2016; Shtulman and Lindeman, 2016; Richert et al., 2017). Overall, studies on how people anthropomorphize God have found that people do differentiate between the mental abilities of God and human beings (Heiphetz et al., 2016). Moving beyond the psychological domain, research has also shown that people attribute more anthropomorphic psychological properties to God than biological or physical properties (Shtulman, 2008; Shtulman and Lindeman, 2016). Finally, research on the contextual factors that might predict individual differences have shown that people anthropomorphize God less when they have more religious exposure, and less when they are from the Islamic religious tradition (Richert et al., 2016, 2017). However, research has yet to fully characterize the underlying structure of anthropomorphic reasoning when it’s applied to concepts of God. Thus, the current study examines (a) the conceptual structure of anthropomorphic reasoning about God, (b) predictors of that structure in individuals, and (c) consistency within and between the sub-domains of that structure in individuals.

### Conceptual Structure of Anthropomorphic Reasoning

The first research question of the current study regards the underlying conceptual structure of anthropomorphic reasoning about God in adults. Adults and children do vary in what human-like properties they attribute to different categories of non-human entities (Waytz et al., 2010; Severson and Lemm, 2016). However, researchers commonly collapse the underlying sub-categories of anthropomorphism together (e.g., animal and non-animal) to create a trait-like score of an individual’s tendency to anthropomorphize. Research has yet to fully explain variation within and across dimensions of anthropomorphic reasoning.

As mentioned above, there is still debate as to whether anthropomorphic reasoning is just the application of agency and mentalizing (i.e., folk-psychological reasoning) to non-human entities, or the application of the entire ‘human’ concept (i.e., all three folk domains). Within concepts of God specifically, adults are more likely to attribute psychological properties to God than physical or biological properties (Shtulman, 2008; Shtulman and Lindeman, 2016). If anthropomorphic reasoning was just the application of agency and mentality, attributions of God’s psychological properties would be unrelated to the attributions of God’s physical and biological properties. The examination of mean differences between domains (psychology versus biology versus physics) has not outlined how or if domains relate to each other within individuals. Thus, the primary goal of the present study was to characterize the underlying structure of anthropomorphic reasoning in individual concepts of God.

**FIGURE 1:**
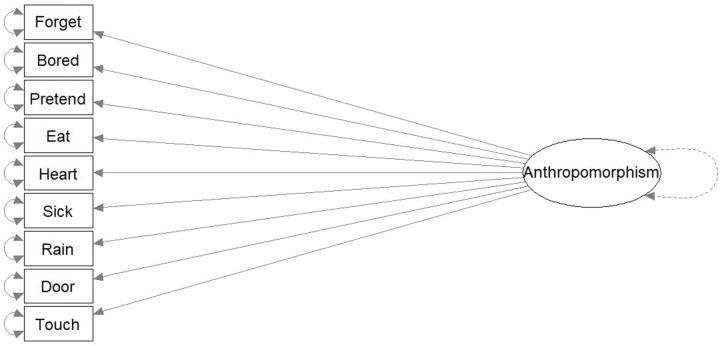
“One-Dimension Concept.” The “One-Dimension Concept” is a theoretical model predicting that the structure of anthropomorphism is one overarching dimension, without sub-domains.

To answer this question, three competing hypotheses were tested. The first hypothesis proposed that there is one overarching dimension of anthropomorphic reasoning, without sub-domains (**Figure 1**). In this structure, an individual who highly anthropomorphizes God in biological traits also would highly anthropomorphize God in psychological and physical traits. This hypothesized structure is labeled as the “One-Dimension Concept.”

**FIGURE 2:**
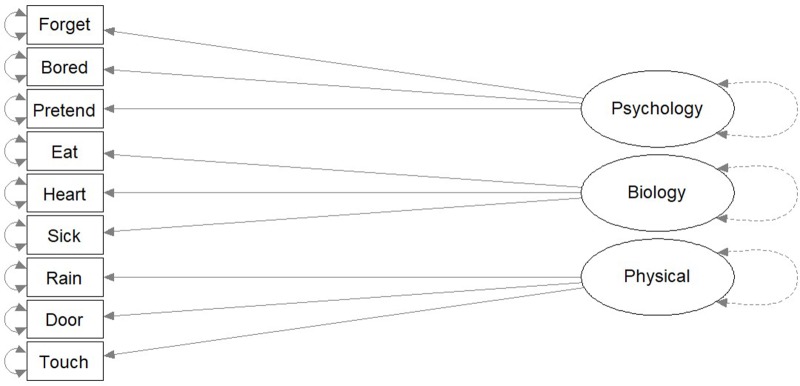
“Independent Dimensions Concept.” The “Independent Dimensions Concept” is a theoretical model predicting that the structure of anthropomorphism is three unrelated dimensions.

The second hypothesis proposed that there are three independent dimensions of anthropomorphic reasoning: psychological, biological, and physical (**Figure 2**). In this structure, an individual’s anthropomorphic reasoning about God in the biological domain would be unrelated to their anthropomorphic reasoning about God in any of the other domains. This hypothesized structure is labeled as the “Independent Dimensions Concept.”

**FIGURE 3:**
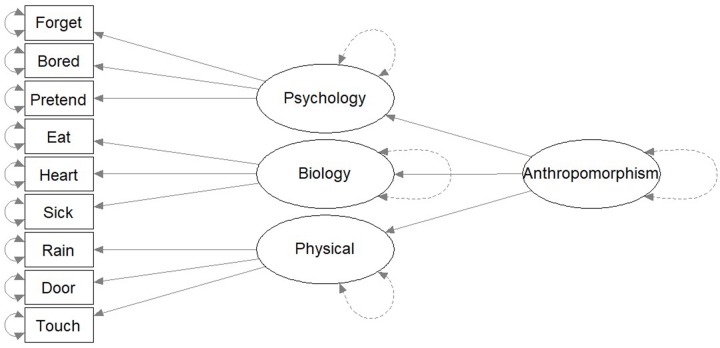
“Hierarchical Dimensions Concept.” The “Hierarchical Dimensions Concept” is a theoretical model predicting that the structure of anthropomorphism is one overarching domain with three sub-domains.

The third hypothesis proposed that there is an overall dimension of anthropomorphic reasoning that is composed of three sub-domains – psychological, biological, and physical – each of which contributes differentially to the overall anthropomorphic concept of God (**Figure 3**). In this structure, an individual’s anthropomorphic reasoning about God in one domain would be partially determined by a domain-general tendency toward anthropomorphizing God while also being independently determined by domain-specific anthropomorphizing of God along a specific dimension. This structure is labeled as the “Hierarchical Dimensions Concept.”

### The Role of Religious Belief and Experience

The second research question was related to understanding what cultural inputs are potential causes of variation between and within individuals regarding their anthropomorphic reasoning about God. Some research suggests that more religious individuals attribute more anthropomorphic properties (e.g., can hear, be aware of things) to God than less religious individuals (Shtulman and Lindeman, 2016). However, other research indicates that the tendency to anthropomorphize in general is unrelated to belief in God (Willard and Norenzayan, 2013). Developmental research in children show that anthropomorphic reasoning about God is unrelated to the frequency of participation in religious practices, instead children’s anthropomorphic reasoning is related to their parents’ anthropomorphic reasoning (Richert et al., 2016). Thus, the present study explored whether religious behavior and/or belief was related to an individual’s anthropomorphic concept of God.

### Sub-domain Consistency of Anthropomorphic Attributions

The third research question was more exploratory and addressed the extent to which people’s anthropomorphic reasoning varies within themselves, between domains in attributing anthropomorphic properties. In other words, people may rate God (or other non-human entities) as highly anthropomorphic across domains or may rate God as highly anthropomorphic in some domains (e.g., psychological) and not others (e.g., biological). Previous research has examined how people differ from one another in their global attributions of anthropomorphism (Waytz et al., 2010; Severson and Lemm, 2016) and in mean differences in the attribution of anthropomorphic properties in domains of anthropomorphism (Shtulman, 2008; Shtulman and Lindeman, 2016). However, it remains unclear where variation exists within people, between domains (psychological versus biological), and potentially within a single domain. Thus, the present study examined if individuals were consistent in their anthropomorphic attributions to God within each domain.

## Materials and Methods

The present study assessed adults’ anthropomorphic concepts of God, the underlying dimensional structure of anthropomorphic reasoning, predictors of the dimensional structure, and the consistency of individuals’ attributions. Adults, varying in religious affiliation, indicated their certainty that God had biological, psychological, and physical attributes. Participants also answered questions about their religious behavior and religious belief.

### Participants

Three hundred and forty-one undergraduate students participated in this study. All participants were recruited through introductory psychology classes at a large university in Southern California. All participants received course credit for participation, and all participants spoke English. Participants had a range of religious affiliations (**Table 1**).

**Table 1:** Age, gender, and religious affiliation of participants.

	Gender		
	*N*	*M*	*SD*
Male	129	19.54	1.71
Female	212	19.49	1.83

	**Religious affiliation**		
	***N***	***M***	***SD***

Protestant Christian	93	19.42	1.61
Roman Catholic	98	19.57	2.05
Muslim	34	19.06	1.07
Non-Affiliated	98	19.67	1.91
Other	18	19.65	1.27
Total	341	19.51	1.78

### Assessments

#### Anthropomorphic Reasoning

Participants answered nine questions about the anthropomorphic properties of God. Of the nine questions, three focused on God’s psychological properties, three focused on God’s biological properties, and three focused on God’s physical properties (see **Table 2** for exact questions). Previous research with adults and children has indicated these nine questions reliably predict anthropomorphic reasoning about God (Richert et al., 2016; Shaman et al., 2016). Participants rated their certainty that God had each of these anthropomorphic properties on a 5-point scale from “Definitely No” [-2] to “Definitely Yes” [+2]).

**Table 2:** Questions assessing anthropomorphic properties of God.

Psychological	Could God forget things?
	Could God get bored?
	Could God have a pretend friend?
Biological	Does God need to eat food and drink water?
	Does God have a heart that keeps God alive?
	Could God get sick?
Physical	Could God get wet when it rains?
	Does God have to open a door to go through it?
	Could you touch God with your hand?


For each domain of anthropomorphic properties, the mean of participants’ ratings was calculated. Thus, each participant had three domain average scores (psychological, biological, and physical) ranging from -2 to +2; a high score indicated the participant thought God was anthropomorphic in that domain and a low score indicated the participant thought God was non-anthropomorphic in that domain. An overall anthropomorphic reasoning score was also calculated by averaging all nine responses (**Table 3**).

**Table 3:** Anthropomorphism of God.

	**M**	**SD**	Cronbach’s α
Psychological	-0.53	1.03	0.655
Biological	-0.64	1.13	0.799
Physical	-0.38	1.04	0.655
Overall	-0.52	0.87	0.828


For each domain of anthropomorphic properties, the standard deviation of participants’ ratings was calculated. Thus, each participant had three domain consistency scores (psychological, biological, and physical); a high score indicated the participant was not consistent in attributing to God anthropomorphic properties within that domain and a low score indicated the participant was consistent in attributing to God anthropomorphic properties within that domain. An overall consistency score was also calculated (**Table 4**).

**Table 4:** Consistency of anthropomorphism ratings.

	**M**	**SD**
Psychological	0.84	0.71
Biological	0.62	0.71
Physical	0.83	0.73
Overall	1.00	0.53

#### Religious Belief

Participants answered questions about their belief in God and belief in the soul, as well as questions about how religious and spiritual they considered themselves to be. Participants indicated their certainty that God and the soul existed on a 5-point scale from “Definitely does not exist” [-2] to “Definitely does exist” [+2]. Overall, participants were somewhat certain that God existed and that the soul existed (**Table 5**). Participants also indicated how religious and spiritual they considered themselves to be compared to the average American on a 5-point scale from “Not at all” [1] to “Very” [5]. Overall, participants considered themselves about average on religiosity and spirituality.

**Table 5:** Religious belief.

	**M**	**SD**
Belief in God	0.99	1.19
Belief in the soul	1.33	0.82
Religiosity	2.84	1.13
Spirituality	3.11	1.14

#### Religious Behavior

Participants answered three questions about the frequency of their religious behavior: attendance at events sponsored by a religious organization (e.g., youth group), engaging in private religious practices (e.g., prayer), and participation in public religious practices (e.g., religious services). Participants rated the frequency of participation on a 9-point scale from “Never” [0] to “Multiple times a day” [8]. These scores were averaged for an overall religious behavior variable. Overall, religious behavior was low, around ‘multiple times a year’ (**Table 6**).

**Table 6:** Frequency of religious behavior.

	**M**	**SD**	Cronbach’s α
Events	2.29	1.98	
Public practices	1.96	2.04	
Private practices	2.84	2.81	
Religious behavior	2.36	1.98	0.82

#### Religious Experiences

Participants also answered questions about their experiences with specific religious activities, rituals, and events. For each event, participants indicated if they experienced it, prayed or observed someone praying during the activity, attended a religious institution for that activity, whether a religious figure was present, and whether they learned about the activity’s meaning. For each aspect of each activity, participants indicated “yes” [1] or “no” [0]. For each question type, the sum of all experiences was calculated (**Table 7**).

**Table 7:** Sum of religious experiences.

	**M**	**SD**	Cronbach’s α
Experienced	9.83	5.08	0.89
Prayed	9.54	5.76	0.92
Religious institution	8.99	5.80	0.92
Religious figure	9.36	6.00	0.93
Meaning	10.97	5.93	0.93


Christian, Catholic, and Non-Affiliated participants responded to 20 activities: Baptism, Christmas, Communion, Easter, Funerals, Lent, Marriage, Pentecost, Last Rites, Bible Study, Confession, Confirmation, Eucharist, Gospel Singing, the Lord’s Prayer, Missionary work, Ordination, the Rosary, making a Pilgrimage, and Speaking in Tongues. Muslim participants responded to 20 different activities: Aqiqa/circumcision, Eid Adha, Eid Fitr, Eid Mubahila, Eid Zehra, Jumah (Friday) Prayer, Muharram/Ashura, Mahe Ramadhan, Wiladat/Shahadat, Qur’an recitation, Namaz/Salah, fasting, Hajj/Umrah, Ziyarat, learning Arabic, attending madressah/Sunday school, learning Fiqh and Hadith, majalis/matam, dua recitation, and praying tasbeeh.

### Procedure

Participants answered the survey electronically over the internet. Participants answered questions about their anthropomorphic concept of God first, followed by the questions about their religious beliefs and behavior. The survey took participants 38.5 min on average to complete.

## Results

### Conceptual Structure of Anthropomorphic Reasoning

The first research question was about the underlying structure of an individual’s anthropomorphic concept of God. Three confirmatory factor analyses (CFA) were conducted to test the three competing hypotheses. For each CFA model, the nine questions assessing different anthropomorphic properties of God were entered as the observed variables (see **Table 8** for correlations between observed variables). Models were estimated using maximum likelihood estimation. Additionally, for each model, the variances of the latent factors were set to 1. Factor loadings were then standardized.

**Table 8:** Correlations between anthropomorphic properties of God questions.

Properties	1	2	3	4	5	6	7	8	9
1. Forget	–								
2. Bored	0.38^∗∗^	–							
3. Pretend	0.29^∗∗^	0.50^∗∗^	–						
4. Eat	0.32^∗∗^	0.25^∗∗^	0.34^∗∗^	–					
5. Heart	0.20^∗∗^	0.22^∗∗^	0.32^∗∗^	0.57^∗∗^	–				
6. Sick	0.39^∗∗^	0.37^∗∗^	0.45^∗∗^	0.63^∗∗^	0.53^∗∗^	–			
7. Rain	0.26^∗∗^	0.29^∗∗^	0.41^∗∗^	0.50^∗∗^	0.44^∗∗^	0.55^∗∗^	–		
8. Door	0.28^∗∗^	0.24^∗∗^	0.34^∗∗^	0.62^∗∗^	0.47^∗∗^	0.54^∗∗^	0.57^∗∗^	–	
9. Touch	0.04	-0.01	0.13^∗^	0.26^∗∗^	0.30^∗∗^	0.19^∗∗^	0.32^∗∗^	0.29^∗∗^	–

*^∗^*p* < 0.05, ^∗∗^*p* < 0.01.*

When fitting data to a model in a CFA, a comparative fit index (CFI) greater than or equal than 0.95, a root mean square error of approximation (RMSEA) less than 0.08, and a standardized root mean square residual (SRMR) less than 0.08 indicate that the model acceptably fits the data (Schreiber et al., 2006). When fit to the data, the one-dimension model did not meet acceptable standards, χ^2^(27) = 144.64, *p* < 0.001, CFI = 0.847, RMSEA = 0.113, SRMR = 0.071. When fit to the data, the independent model did not meet acceptable standards, χ^2^(27) = 397.842, *p* < 0.001, CFI = 0.638, RMSEA = 0.201, SRMR = 0.274. When fit to the data, the hierarchical model did meet acceptable standards, χ^2^(24) = 73.086, *p* < 0.001, CFI = 0.952, RMSEA = 0.077, SRMR = 0.051.

An additional way to assess model fit is to compare models to each other rather than assessing if each model meets acceptable standards. When using Akaike information criterion (AIC), smaller values indicate the model fits the data better than larger values (Schreiber et al., 2006). Not only was the “hierarchical dimensions concept” model the only one to acceptably meet the fit criteria, when using a comparative fit statistic, the hierarchical model (AIC = 9543.567) fit the data better than the one-dimension model (AIC = 9609.125), which fit the data better than the independent model (AIC = 9862.323).

The findings from the three CFA analyses support the “hierarchical dimensions concept” hypothesis. In other words, when individuals conceptualize God’s anthropomorphic properties, they conceptualize the psychological, biological, and physical properties differently from one another; however, each sub-domain of properties is influenced by superordinate anthropomorphic reasoning about God.

#### Sub-domain Analysis

Given the data suggest the hierarchical dimensions model best fit the data, a deeper examination of the sub-domains and the relations between them was warranted. As seen in **Table 9**, the CFA indicated the biological sub-domains loaded more strongly onto the latent construct of anthropomorphic reasoning about God than the psychological and physical sub-domains.

**Table 9:** Unstandardized and standardized coefficients for hierarchical dimensions model.

Observed variables	Latent construct	β	*B*	*SE*
Forget	Psychological	0.52	0.51	0.07
Bored	Psychological	0.67	0.66	0.07
Pretend	Psychological	0.68	0.71	0.07
Eat	Biological	0.08	0.80	0.58
Heart	Biological	0.07	0.67	0.51
Sick	Biological	0.08	0.80	0.52
Rain	Physical	0.42	0.74	0.09
Door	Physical	0.45	0.79	0.10
Touch	Physical	0.23	0.37	0.06
**Predictors**				
Psychological	Anthropomorphism	0.89	0.67	0.12
Biological	Anthropomorphism	12.90	0.99	89.44
Physical	Anthropomorphism	2.08	0.90	0.53

For further exploration, a Repeated-Measures ANOVA was conducted examining mean differences between the domains (see **Table 3** for means and standard deviations). There was a significant effect of sub-domain, *F*(2, 680) = 10.018, *p* < 0.001, $\eta_{p}^{2}$ = 0.029. Participants did not rate God’s psychological properties different than God’s biological properties [*t*(340) = 1.770, *p* = 0.78, $\eta_{p}^{2}$ = 0.012]. However, participants did rate God as more anthropomorphic in the physical domain than in the psychological [*t*(340) = 2.390, *p* = 0.038, $\eta_{p}^{2}$ = 0.013] and biological [*t*(340) = 5.223, *p* < 0.001, $\eta_{p}^{2}$ = 0.075] domains.

The analyses of participants’ attributions of anthropomorphic properties to God revealed three primary findings. First, within a concept of God, anthropomorphic reasoning exists as latent, hierarchical construct consisting of three sub-domains: biological, psychological, and physical. Second, participants are more likely to infer God has physical anthropomorphic properties to God than psychological or biological properties. Third, participants’ attribution of biological properties to God contributes more than attributions in other sub-domains to an individual’s overall anthropomorphic concept of God. Because the hierarchical structure of anthropomorphic reasoning in this case (e.g., concept of God) emerged, further analyses examined predictors of variation in participants’ anthropomorphic reasoning within the three sub-domains.

### The Role of Religious Belief and Experience

The second research question asked whether religious behavior and/or belief was related to an individual’s anthropomorphic concept of God. A series of correlations were conducted to assess how participants’ belief, religious behavior, and religious experiences, were related to their mean levels of anthropomorphic reasoning and their mean levels of each sub-domain of anthropomorphic reasoning (**Table 9**).

First, a 3 × 4 Repeated-Measures ANOVA was conducted examining the mean differences between the sub-domains, as a within-subjects variable, and religious affiliation as a between-subjects variable. For this analysis, participants who listed ‘Other” were removed from the analysis due to the small number of participants. There was a significant main effect of domain, *F*(2, 638) = 7.660, *p* = 0.001, $\eta_{p}^{2}$ = 0.023. Again, participants rated God as more anthropomorphic in the physical domain than the psychological and biological domains. There was also a significant main effect of affiliation, *F*(3, 319) = 11.067, *p* < 0.001, $\eta_{p}^{2}$ = 0.094. Bonferroni *post hoc* tests indicated that Protestant Christian and Muslim participants significantly anthropomorphized God less than the Roman Catholic and Non-Affiliated participants. Finally, there was a significant interaction between domain and religious affiliation, *F*(6, 638) = 8.811, *p* < 0.001, $\eta_{p}^{2}$ = 0.077.

**FIGURE 4:**
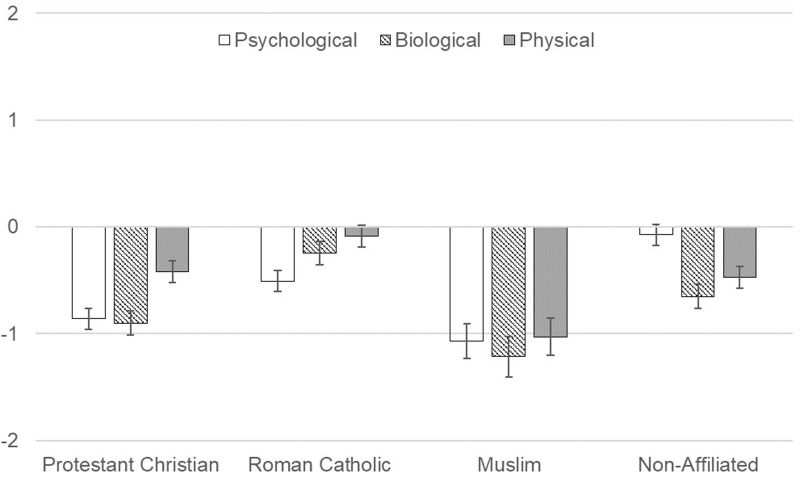
Anthropomorphism of God by sub-domain and religious affiliation. Mean levels of anthropomorphic attributions to God within each sub-domain separated by religious affiliation.

Follow-up one-way ANOVAs were conducted to compare religious affiliations among each domain (**Figure 4**). For the psychological domain, the affiliations did significantly differ, *F*(3, 319) = 14.441, *p* < 0.001, $\eta_{p}^{2}$ = 0.120. Bonferroni adjusted *post hoc* tests indicated Non-Affiliated participants anthropomorphized God significantly more than the other three religious affiliations. Roman Catholic participants did not differ from Protestant Christians but did anthropomorphize God significantly more than Muslim participants. For the biological domain, the affiliations did significantly differ, *F*(3, 319) = 9.135, *p* < 0.001, $\eta_{p}^{2}$ = 0.079. Bonferroni adjusted *post hoc* tests indicated Roman Catholic participants anthropomorphized God significantly more than Protestant Christian and Muslim participants, but not Non-Affiliated participants. No other significant differences existed among religious affiliations. For the physical domain, the affiliations did significantly differ, *F*(3, 319) = 7.790, *p* < 0.001, $\eta_{p}^{2}$ = 0.068. Bonferroni adjusted *post hoc* tests indicated Muslim participants anthropomorphized God significantly less than all other religious affiliations. Roman Catholic participants anthropomorphized God significantly more than Non-Affiliated participants.

In summary, Muslim participants anthropomorphized God less than participations from other religious backgrounds. Regarding the relations of the domains to each other, participants had strongest anthropomorphic reasoning about God in the physical domain, but patterns of psychological and biological anthropomorphic reasoning differed by religious background. Among Protestant Christian, Muslim, and Non-Affiliated participants, God was anthropomorphized least in the biological domain, with anthropomorphic reasoning in the psychological domain falling between the physical and biological domains. However, for the Roman Catholic participants, God was anthropomorphized least in the psychological domain, with the biological domain between the psychological and physical domains.

Beyond identification with a specific religious affiliation, a pattern emerged in how participants’ religious behavior and belief were related to their anthropomorphic reasoning about God (**Table 10**). No significant correlations emerged between participants’ biological or physical anthropomorphizing and their beliefs or experiences. However, participants who had higher levels of belief in God and the soul, reported stronger religiosity and spirituality, greater frequency of religious behavior, and greater frequency of participation in religious practices were less likely to anthropomorphize God in the psychological domain.

**Table 10:** Correlations between anthropomorphism and religious belief and behavior variables.

	Psychological	Biological	Physical	Overall
Belief				
God	-0.35^∗∗^	-0.08	-0.05	-0.19^∗∗^
Soul	-0.35^∗∗^	-0.13^∗^	-0.10	-0.23^∗∗^
Religiosity	-0.29^∗∗^	0.01	0.04	-0.09
Spirituality	-0.29^∗∗^	-0.07	0.00	-0.14^∗∗^
Behavior	-0.39^∗∗^	-0.17^∗∗^	-0.11^∗^	-0.27^∗∗^
Religious experiences				
Experience	-0.21^∗∗^	0.01	0.01	-0.08
Observe	-0.25^∗∗^	-0.02	0.02	-0.10
Attend	-0.24^∗∗^	-0.01	0.01	-0.10
Religious figure	-0.20^∗∗^	-0.01	0.03	-0.07
Learned meaning	-0.21^∗∗^	-0.05	-0.02	-0.11^∗^

*^∗^*p* < 0.05, ^∗∗^*p* < 0.01.*

Thus, the present study added evidence to support the hypothesis that religious beliefs and behavior are related to participants’ anthropomorphic reasoning of God (Shtulman and Lindeman, 2016). However, the findings are more nuanced. Religious beliefs and experiences are specifically related to how participants conceptualized the psychological properties of God, but not God’s biological and physical properties.

### Sub-domain Consistency of Anthropomorphic Attributions

The third research question asked whether individuals were consistent in their anthropomorphic attributions to God within each domain. A Repeated-Measures ANOVA was conducted to determine if participants differed in how consistent they were in their anthropomorphic attributions of God between each domain. Additionally, a series of correlations were conducted to assess how participants’ belief, religious behavior, and religious experiences were related to their consistency of anthropomorphism scores.

First, a 3 × 4 Repeated-Measures ANOVA was conducted examining the consistency of responses within each sub-domain, as a within-subjects variable, and religious affiliation as a between-subjects variable. For this analysis, participants who listed ‘Other” were removed from the analysis due to the small number of participants. There was a significant main effect of domain, *F*(2, 638) = 9.822, *p* < 0.001, $\eta_{p}^{2}$ = 0.030. Participants were more consistent (i.e., varied less) in their responses in the biological domain than the psychological [*t*(340) = 4.601, *p* < 0.001, $\eta_{p}^{2}$ = 0.059] or physical domains [*t*(340) = 4.209, *p* < 0.001, $\eta_{p}^{2}$ = 0.052]. Participants did not differ in their consistency within the psychological or physical domains [*t*(340) = 0.149, *p* = 0.88, $\eta_{p}^{2}$ < 0.001].

**FIGURE 5:**
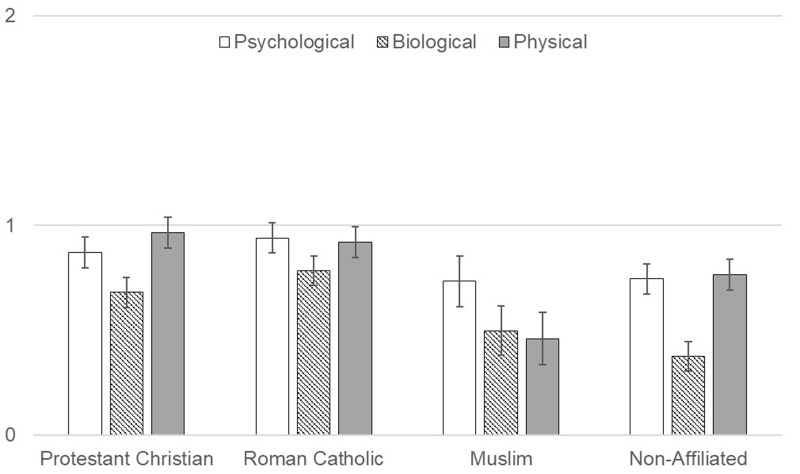
Consistency of anthropomorphism of God by sub-domain and religious affiliation. Consistency of anthropomorphism attributions to God within each sub-domain separated by religious affiliation.

As with mean levels of anthropomorphic reasoning in the different sub-domains, there was also a significant main effect of affiliation, *F*(3, 319) = 7.849, *p* < 0.001, $\eta_{p}^{2}$ = 0.069. The nature of this interaction, however, was different than that for mean levels. Bonferroni *post hoc* tests indicated that Protestant Christian and Roman Catholic participants were significantly less consistent in their attributions of anthropomorphic properties than Muslim and Non-Affiliated participants (**Figure 5**). Finally, there was no significant interaction between domain and religious affiliation, *F*(6, 638) = 1.553, *p* = 0.158, $\eta_{p}^{2}$ = 0.014.

As seen in **Table 11**, a pattern emerged in how participants’ religious behavior and belief were related to their consistency in anthropomorphizing God that was different from the pattern with mean levels of anthropomorphic reasoning. Participants who had higher levels of belief/religiosity, as well as higher levels of religious behavior and experiences, were less consistent in how they anthropomorphized God in the biological domain. These possible predictors of variation in participants’ anthropomorphic reasoning of God were unrelated to participants’ consistency in the psychological and physical domains.

**Table 11:** Correlations between consistency of anthropomorphism and religious belief and behavior variables.

	Psychological	Biological	Physical	Overall
Belief				
God	0.11^∗^	0.19^∗∗^	0.03	0.18^∗∗^
Soul	0.09	0.16^∗∗^	0.14^∗^	0.21^∗∗^
Religiosity	-0.08	0.14^∗∗^	0.02	0.09
Spirituality	-0.02	0.13^∗^	0.04	0.11^∗^
Behavior	-0.05	0.13^∗^	0.01	0.07
Religious experiences				
Experience	0.03	0.15^∗∗^	0.04	0.11
Observe	0.02	0.14^∗^	0.03	0.10
Attend	0.02	0.12^∗^	0.04	0.10
Religious figure	0.05	0.13^∗^	0.02	0.11
Learned meaning	0.04	0.14^∗^	0.07	0.15

*^∗^*p* < 0.05, ^∗∗^*p* < 0.01.*

Thus, the present study indicated that the biological sub-domain had greater consistency as compared with the other domains; and the consistency of the biological sub-domain was related to individuals’ religious beliefs and experiences.

## Discussion

The present study sought to determine the underlying structure of the anthropomorphic concept of God, whether religious belief and/or religious behavior were related to that structure, and whether individuals were consistent in their anthropomorphic concept of God. Participants indicated their certainty that God had nine anthropomorphic properties that fell within three sub-domains: biological, psychological, and physical. Participants also provided details about their religious beliefs and behavior. Confirmatory factor analyses were conducted to assess the structure of the anthropomorphic concept of God, and correlations and Repeated-Measures ANOVAs were conducted to assess which predictors were associated with participants’ anthropomorphism of God. Findings are discussed as they relate to the specific research questions regarding: (a) the conceptual structure of anthropomorphic reasoning about God, (b) predictors of variation in individuals’ anthropomorphic reasoning about God, and (c) individual consistency of anthropomorphizing within biological, psychological, and physical domains.

### Conceptual Structure of Anthropomorphic Reasoning

In order to address the first research question, which asked about the underlying structure of an individual’s anthropomorphic concept of God, three hypotheses were proposed. The “One Dimension Concept” hypothesis suggested that there was one overall dimension of anthropomorphic reasoning that dictated how a person viewed all of God’s anthropomorphic properties. The “Independent Dimensions Concept” hypothesis suggested that there were three independent domains of anthropomorphic reasoning and how an individual viewed God’s anthropomorphic properties in one domain was unrelated to the other domains. The “Hierarchical Dimensions Concept” hypothesis suggested that there was one overall dimension of anthropomorphic reasoning with three sub-domains and how an individual viewed God’s anthropomorphic properties in one domain was both related to an overall concept of anthropomorphic reasoning as well as an individual concept in that domain.

Three CFAs were conducted testing each hypothesis and the data fit the hierarchical model the best supporting the Hierarchical Dimensions Concept hypothesis. This finding suggests that when individuals anthropomorphize God, they make domain-specific inferences about God’s psychological, biological, and physical properties, but each domain is additionally influenced by an overarching concept of anthropomorphism that causes the domains to relate to one another.

One possible interpretation of this finding is that people may use their overall concept of ‘human’ to reason about God rather than just their concepts of agency and mentalizing. If people only used folk-psychological reasoning in their concept of God, then the psychological domain would be the primary, if not only, contributor to their overall anthropomorphic concept of God. Instead, the results of the CFA suggested that people’s biological concept of God contributed most strongly their overall anthropomorphic concept of God. One thing to consider is that by merely asking participants about God’s psychological, biological, and physical traits, we prime them to use those domains of folk reasoning to answer the questions in the way that they did (i.e., this is a methodological artifact). However, the existence of a super-ordinate dimension that caused each of the sub-domains to be related to one another suggests that the more likely explanation is that people do apply their concept of ‘human’ to God.

To some extent, these findings also further support those of Waytz et al. (2010) and Shtulman and Lindeman (2016), in that both studies demonstrated evidence of dispositional or trait-level differences in anthropomorphizing. The fact that, in this study, the underlying dimensions of psychological, biological, and physical attributions all contributed to the same latent construct indicate that there is a general tendency toward or against anthropomorphizing that contributes to an individual’s attribution of anthropomorphic traits to God in all three domains.

However, the current findings also extend both Waytz et al. (2010) and Shtulman and Lindeman’s (2016) findings in meaningful ways. Waytz et al. (2010) indicated the IDAQ resulted in three constructs (animals, non-animals, and spiritual beings). In regards to animals and non-animals, participants readily distinguished the anthropomorphic and non-anthropomorphic characteristics (Waytz et al., 2010). However, participants responded similarly to the anthropomorphic and non-anthropomorphic characteristics of spiritual beings. Waytz et al. (2010) suggested that anthropomorphic responses about spiritual beings reflected belief in or recognition of spiritual beings, rather than anthropomorphic reasoning. The finding in the present study that belief in God was related to psychological attributions to God, not biological or physical attributions, suggests that participants’ anthropomorphic attributions to God (or other spiritual beings) may not serve as a proxy for belief, but may instead reflect domain-level distinctions in which human-like characteristics God does and does not have. This interpretation is further supported by the fact that Waytz et al. (2010) asked participants about attributions of mentalizing and agency but did not inquire about biological or physical attributions.

The findings regarding the sub-domains of anthropomorphism also may appear to contrast with Shtulman and Lindeman (2016), particularly in the fact that Shtulman and Lindeman (2016) found participants indicated God had more psychological anthropomorphic attributes than physiological attributes. In contrast, participants in the current study indicated God had more physical anthropomorphic attributes than biological or psychological attributes. One reason for the difference may be that Shtulman and Lindeman (2016) combined biological and physical attributes into an overall physiological domain. An additional reason for the difference may be the current study assessed psychological anthropomorphic properties in a different way. Shtulman and Lindeman (2016) assessed psychological attributes that closely matched the basic concepts of agency and mentalizing, including properties of beliefs, desires, intentions, emotions, and perceptions. However, the current study assessed psychological attributes that were more human-like. Given that the current study looked at more uniquely human attributes, participants may have been less likely to apply them to God. In other words, individuals may be more likely to attribute agency-related psychological attributes to God than human-related psychological attributes to God.

These findings suggest the importance of differentiating general attributions of agency from more specific attributions of humanness in studies of anthropomorphism. The current study was not designed to test between these forms of anthropomorphism, but the hierarchical structure of anthropomorphism to God suggests two possibilities. One possibility is that the hierarchical structure of anthropomorphism of God reflects the logical, structural relation between the three domains of folk knowledge. An individual’s use of their folk psychological reasoning may trigger their use of folk biological and folk physical reasoning. If an entity has mental states, then it carries that the entity also has a biological and physical body to support those mental states. In the case of the current study, the latent construct of overall anthropomorphic reasoning would then reflect the expression of this inference. If individuals attribute any psychological properties to God, they would be more likely to attribute biological and physical properties as well.

A more likely possibility, given the overall patterns in the results, is that participants used their concept of ‘human’ and of human limitations to reason about all aspects of God, hence the latent anthropomorphic domain was that of ‘human.’ In particular, participants inferred human-like physical characteristics to God more than psychological characteristics. Additionally, the biological domain loaded more strongly on the superordinate domain than the psychological domain. When reasoning within these domains, then, participants likely were relying on their concept of ‘human’ to make inferences about God; the concept of ‘human’ would then lead to reasoning in each of the folk domains, hence the sub-domains in the model.

### The Role of Religious Belief and Experience

The second research question asked whether an individual’s anthropomorphic concept of God was related to their religious beliefs, behavior, and/or experiences. Participants’ self-reported religious beliefs, behaviors, and experiences were correlated with their attributions of anthropomorphic properties to God in each sub-domain. Overall, participants’ religious beliefs, behaviors, and experiences were significantly and negatively related to their anthropomorphic concept of God in the psychological domain, but not the biological or physical domains. When participants believed in God and the soul, had higher religiosity and spirituality, engaged in more religious behaviors, and had more religious experiences, they attributed less anthropomorphic psychological properties to God. In other words, when participants were more engaged in their religion overall, they did not think of God as having human-like psychological characteristics. These findings are in contrast with those of Shtulman and Lindeman (2016), who found that religiosity was positively related to an anthropomorphic concept of God. As discussed above, the different results may be due to the differences in how the psychological properties of God were assessed, and due to the fact that Shtulman and Lindeman (2016) combined the biological and physical domains.

The interactions between domain and religious affiliation further indicate that anthropomorphic reasoning about God exists along related but different dimensions. As in previous studies, participants who affiliated with Islam were less likely than other participants to anthropomorphize God (Richert et al., 2017). Additionally, Protestant Christian, Muslim, and Non-Affiliated participants had lowest anthropomorphic reasoning in the biological domain, with anthropomorphic reasoning in the psychological domain falling between the physical and biological domains. In contrast, the Roman Catholic participants had lowest anthropomorphic reasoning about God in the psychological domain.

The relationship between religious beliefs and experiences and anthropomorphic reasoning about God provides further support for the “Hierarchical Dimensions Concept” model discussed above. If individuals only had one overall anthropomorphic concept of God, then their religious beliefs and experiences would be related to all of the attributes of God. However, as described above, biological, psychological, and physical inferences represent distinct sub-domains of anthropomorphic reasoning. In the current study, engagement in religion was only related to psychological anthropomorphic reasoning, suggesting that concepts of God’s agency or psychological anthropomorphic reasoning overlap strongly with belief in God (i.e., Waytz et al., 2010). Understanding psychological anthropomorphic reasoning as a distinct, yet related dimension of anthropomorphism is further supported by findings indicating the social-cognitive nature of religious cognition (Richert and Smith, 2009; Rottman and Kelemen, 2012). Research on prayer (Spilka and Ladd, 2013; Shaman et al., 2016), rituals (Richert, 2006; Watson-Jones and Legare, 2016), and afterlife beliefs (Bering et al., 2005) has found evidence that reasoning about these religious concepts utilizes an individual’s social cognition. Social cognition refers to the aspects of cognition dedicated to how humans understand themselves and other agents in the context of a social environment; and so the connection between these cultural phenomena and social cognition suggests that cultural inputs would more strongly influence thinking about the *mind* of God (i.e., utilizing social cognition and folk psychology) as opposed to the biological and physical aspects of God’s body (i.e., physical and biological cognition).

The findings of the current study suggest there are unique differences in how people reason about the biological and psychological properties of God. The biological sub-domain contributed the most strongly to the superordinate anthropomorphism concept, offering further support for the hypothesis that the latent anthropomorphism construct is more strongly related to anthropomorphizing based in ‘humanness’ rather than ‘agency.’

### Sub-domain Consistency Anthropomorphic Reasoning

The “Hierarchical Dimensions Concept” is further supported by findings regarding different levels of consistency in the different domains of anthropomorphizing God. Consistency in item ratings was lower in the biological sub-domain than in the physical and psychological sub-domain. Additionally, although consistency did not vary by religious affiliation, participants’ religious beliefs, behaviors, and experiences were significantly and positively related to their consistency in anthropomorphizing God in the biological domain, but not the psychological or physical domains. When participants believed in God and the soul more, had higher religiosity and spirituality, engaged in more religious behaviors, and had more religious experiences, they were less consistent in thinking about the biological properties of God. In other words, when participants were more engaged in their religious overall, they were more variable in how they thought about the human-like biological properties of God. As with the mean levels of anthropomorphic reasoning, if individuals only had one overall anthropomorphic concept of God then religious beliefs and experiences would be related to consistency in all three domains. The differences in consistency, however, suggest that distinct sub-domains of anthropomorphic reasoning exist.

### Developmental Implications

The current findings point to the need for research into the development and coordination of hierarchical anthropomorphic reasoning in concepts of God. Concepts of God do not emerge spontaneously in adulthood, and children tend to anthropomorphize God in general, more than adults do (Shtulman, 2008; Richert et al., 2016). Reasoning about God, including in some cases the attribution of anthropomorphic psychological, biological, and physical properties, begins as early as 3 or 4 years old (Lane et al., 2012; Richert et al., 2016). However, recent findings indicate children’s anthropomorphism of God differs by religious tradition; as with the current study with Muslim adults, Muslim children did not attribute physical, biological, and psychological human-like traits to God (Richert et al., 2016).

Additionally, theoretical debates exist as to whether younger children do or do not anthropomorphize God’s mind (Barrett and Richert, 2003; Lane and Harris, 2014; Richert et al., 2017). Early in development, children tend not to differentiate the mental abilities of God and human beings. But as children grow older, the difference between the two types of minds increases; God is conceptualized as less and less constrained by the limitations that the human mind has. Recent findings suggest the ages at which children differentiate God’s mind from human minds (Richert et al., 2017) and whether children attribute human-like limitations to God’s mind at some developmental time period (Lane et al., 2014) are related to children’s religious context and their parents’ beliefs.

Examining children’s tendency to anthropomorphize non-human entities, Severson and Lemm (2016) adapted the IDAQ for use with children. The IDAQ-CF modified the questions in the original questionnaire to be comprehensible to young children and found a similar factor structure and individual differences among children. Overall, children were more likely to anthropomorphize animals than non-animal entities. And while there were no overall age trends, older children were more likely to anthropomorphize animals than younger children. Other developmental research has explored the sociocultural influences on anthropomorphizing God and differentiating God’s mind from humans’ minds; influences such as what religious tradition the individual is being raised in, their level of religious exposure, and their parents beliefs about God (Richert et al., 2016, 2017).

The current study helps to shed light on two debates in developmental research on anthropomorphic concepts of God, that of: (a) the underlying nature of children’s supernatural concepts, and (b) the influence of sociocultural factors on children’s anthropomorphic concepts. Two of the central findings in this study–that there is one overall dimension of anthropomorphic reasoning with three sub-domains (i.e., Hierarchical Dimensions), and that religious beliefs and behaviors are related to the psychological sub-domain of anthropomorphic reasoning but not the other two sub-domains–provides direction for future developmental research. Future research should explore whether and how the underlying dimensional structure of anthropomorphic concepts of God changes across development; and at what point in development those religious factors become more or less relevant to reasoning about God.

### Limitations and Future Directions

The current study has certain limitations which suggest directions for future research. One potential limitation in the study was the relative narrow way in which anthropomorphic reasoning about God was assessed. Participants indicated their certainty that God was anthropomorphic in only nine properties. This assessment is a possible reason for the discrepancy between the results of the current study and those of Waytz et al. (2010) and Shtulman and Lindeman (2016). For example, psychological attributes in previous research were strictly related to a general concept of agency, while the attributes in the present study were related more to the specific a concept of ‘human.’ Future research should include a wider range of anthropomorphic properties that assesses both concepts of agency and ‘human’ to see where differences may lie.

A second limitation was the size of the sample. While the sample had an appropriate size for the Repeated-Measures ANOVA analyses and correlations, the size only just met what was necessary for simple CFAs. A more in-depth analysis of the hierarchical structure was not possible without more participants. In particular, a larger sample size would have been better able to address if the conceptual structure differed by religious affiliation or other cultural context variables. Future research should continue to explore the underlying structure of anthropomorphic reasoning and determine if it varies by any other meaningful religious context or cognitive variables. For example, the findings of the present study suggest that Muslim and Non-Affiliated participants are the most consistent in how they conceptualize God’s anthropomorphize properties. A CFA with a larger sample size can assess whether Muslim and Non-Affiliated participants attribute anthropomorphic properties to God using the “Hierarchical Dimensions Concept” or perhaps one of the other models.

## Conclusion

The findings of the present study suggest that individuals have an anthropomorphic concept of God that is hierarchical and composed of three sub-domains. A concept of God is influenced by an overall anthropomorphic concept and separately influenced by sub-domains of anthropomorphic reasoning (i.e., psychological, biological, and physical). For example, when a person thinks about God’s mental properties (i.e., God can forget something), they make inferences based upon two concepts: a superordinate anthropomorphic concept and a subordinate psychological anthropomorphic concept. But when that same person thinks about God’s biological properties (i.e., God can get sick), they make inferences based upon the same superordinate anthropomorphic concept, but a different subordinate concept, biological anthropomorphism.

The presence and use of these sub-domains is important because they are differentially affected by people’s cultural environments. When a person engages more in their religion, they also think of God less anthropomorphically in the psychological domain. But when a person engages more in their religion, they are less consistent in thinking about God’s anthropomorphic properties in the biological domain. The implication is that when studying the tendency to anthropomorphize non-human entities and what influences that tendency, researchers must look deeper. Individuals do not just vary between each other in how they anthropomorphize God but vary within themselves as well.

## Ethics Statement

This study was carried out in accordance with the recommendations of the UCR Human Research Review Board. The protocol was approved by the UCR Human Research Review Board. All subjects gave written informed consent in accordance with the Declaration of Helsinki.

## Author Contributions

NS conducted the analyses and wrote significant portions of the introduction, methods, and results section. All authors equally contributed to the introduction and discussion sections. All authors contributed to the writing of the article and editing of the article.

## Conflict of Interest Statement

The authors declare that the research was conducted in the absence of any commercial or financial relationships that could be construed as a potential conflict of interest.
